# RRHGE: A Novel Approach to Classify the Estrogen Receptor Based Breast Cancer Subtypes

**DOI:** 10.1155/2014/362141

**Published:** 2014-01-19

**Authors:** Ashish Saini, Jingyu Hou, Wanlei Zhou

**Affiliations:** School of Information Technology, Deakin University, 221 Burwood Highway, Melbourne, VIC 3125, Australia

## Abstract

*Background*. Breast cancer is the most common type of cancer among females with a high mortality rate. It is essential to classify the estrogen receptor based breast cancer subtypes into correct subclasses, so that the right treatments can be applied to lower the mortality rate. Using gene signatures derived from gene interaction networks to classify breast cancers has proven to be more reproducible and can achieve higher classification performance. However, the interactions in the gene interaction network usually contain many false-positive interactions that do not have any biological meanings. Therefore, it is a challenge to incorporate the reliability assessment of interactions when deriving gene signatures from gene interaction networks. How to effectively extract gene signatures from available resources is critical to the success of cancer classification. *Methods*. We propose a novel method to measure and extract the reliable (biologically true or valid) interactions from gene interaction networks and incorporate the extracted reliable gene interactions into our proposed *RRHGE* algorithm to identify significant gene signatures from microarray gene expression data for classifying ER+ and ER− breast cancer samples. *Results*. The evaluation on real breast cancer samples showed that our *RRHGE* algorithm achieved higher classification accuracy than the existing approaches.

## 1. Introduction

The diagnosis or prognosis of cancer is believed as one of the most significant research areas in the bioinformatics field. Traditionally, cancer classification is solely based on clinical evidence and requires pathological expertise for biological interpretation. A major challenge in clinical cancer research is the accurate classification of cancers for improving prognosis and treatment. With the rapid development of high-throughput technologies, researchers and biologists have generated a massive amount of data at different levels, such as gene expression profiles using microarrays [1], protein-protein interactions (PPI) [2, 3], gene ontology terms [4], and pathways [5]. These biological data make it possible for biologists and researchers to find solutions to various biological questions of interest, such as the diagnosis of breast cancer by identifying cancer-associated genes.

Due to the increasing use of microarray technology that obtains expression levels of all genes simultaneously, a set of gene expression markers (also known as gene signatures) can be used to diagnose breast cancer in a comprehensive manner [6]. However, existing gene signatures do show variable performances across datasets which makes the classification results unstable [7]. Due to the heterogeneous nature of existing gene signatures, many patients have been classified into the wrong breast cancer subtype and treated with unnecessary adjuvant therapy (chemo or radiation therapy). To solve this problem, various microarray data based breast cancer classification methods have been proposed that use statistical and machine-learning methods for the molecular classification of breast cancer [7–10]. Van de Vijver et al. [11] developed the 70-gene signature (Mammaprint) that classifies breast cancer patients into good or poor prognosis groups. Wang et al. [12] developed a 76-gene signature that consists of 60 genes for the ER+ (estrogen receptor-positive) group and 16 genes for the ER− (estrogen receptor-negative) group in order to classify and to predict the distant metastasis of breast cancer. It was observed that the gene signatures generated in these studies were not robust and heavily depended on the chosen training set [13]. In order to derive the gene signatures from the microarray data and to accurately uncover the molecular forms of breast cancer, plus use the gene signatures for various clinical purposes, the robustness and biological meaning of gene signatures are equally essential [7].

Chuang et al. [14] indicate that a disease like cancer originates from the driver genes that progressively change the expressions of greater amplitude in genes that participate (or interacts) with the driver gene (also called mutations). For the classification of breast cancer, it is therefore good to incorporate the gene network based approach for the following reasons: (1) the gene networks provide models of the molecular mechanisms underlying breast cancer; (2) the detected subnetworks from a gene network are comparatively more reproducible across different breast cancer cohorts than traditional individual genes selected without consideration of network related information; and (3) the gene network based approach achieves higher accuracy in classifying breast cancer subtypes [14].

Various network based approaches have been proposed for microarray data analysis. Gill et al. [15] constructed the condition-dependent networks from differential gene expression with no prior interaction information used (such as PPI or gene regulatory information), which limits the biological validation of their results [7]. Chuang et al. [14] proposed the network based approach that detects differentially expressed subnetworks from the existing PPI data by making use of the local subnetworks aggregation. A network based algorithm (ITI) has been proposed by Garcia et al. [7] that identifies the subnetwork based gene signatures generalizable over multiple and heterogeneous microarray datasets by making use of the PPI data incorporated with the gene expression datasets.

These existing network based approaches address the biological question of interest to some extent. However, these approaches have some issues associated with them, for example: (1) the classifier performance is largely affected by the dataset size [7]; (2) the curse of dimensionality issue (too few samples (in the order of hundreds) for too many genes (in the order of tens of thousands)) is not considered carefully and still needs to be resolved [7]; and, most importantly, (3) the existing PPI datasets such as DIP [16] and HPRD [17] contain many false-positive interactions (i.e., interactions identified by experiments but actually never happen) [18]. Therefore, the existing approaches that use PPI datasets in discovering knowledge or biological facts (such as breast cancer classification, distant metastasis prediction) may be distorted or biased.

The HRGE algorithm [19] tried to address the above issues by identifying hub gene subnetworks and figuring out gene signatures to classify ER+ and ER− breast cancer. However, for HRGE, the reliability weights are empirical when they are incorporated into the algorithm to construct gene signatures. Therefore, the classification results are not stable for some cases. A stable and robust statistic model is required to determine the reliability weights. Furthermore, no independent testing datasets were used when the effectiveness of the HRGE algorithm was evaluated, and no biological validations were conducted as well.

The identification and the extraction of reliable protein interactions from the original experimental PPI datasets is becoming one of the most significant and challenging tasks when using the PPI data for biological analyses. Therefore, to resolve the above issues when classifying cancers, it is essential to develop a novel network based breast cancer classifier which maximises the reliable information for the interactions in the network and is able to provide the optimal classification performance across datasets.

In this paper, we propose a novel subnetwork based breast cancer classification approach to distinguish two subtypes of breast cancer, that is, ER+ and ER−. To increase the sample size of the study and to lessen the dependence on a single training set, we integrated multiple datasets. We used six training gene expression sets on the basis of the histologic grade and the estrogen receptor status, in order to derive the subnetwork based gene signatures, and used two testing gene expression sets for evaluating the performance of gene signatures. We propose a statistical model to determine the reliability weights, which are then incorporated with the gene expressions to form reliable gene expressions. The reliable gene expressions then extracts the subnetworks (isolated networks) and the associated hub-genes (a gene that has a maximum number of interactions in a subnetwork) for the gene signature construction that can be used for the ER+/ER– breast cancer classification paradigm. We call our algorithm robust reliability based hub gene expression (*RRHGE*) algorithm. The evaluation of our approach and the experimental comparisons with other existing approaches demonstrated that *RRHGE* significantly increased the classification performance. Further, in addition to the statistical evaluations, thorough biological evaluations were also conducted to show the effectiveness and stability of the proposed algorithm.

This paper is organized as follows. The training and testing sets used in this study are defined in Section 2. The proposed *RRHGE* algorithm is defined in Section 3. Statistical validation with patient classification results and biological validation are presented in Section 4. Finally, we conclude this paper in Section 5.

## 2. Materials

We downloaded six PPI datasets (BIOGRID, INTACT, MINT, DIP, BIND, and HPRD) and five breast cancer microarray gene expression datasets (GSE7390, GSE6532, GSE21653, GSE11121, and van de Vijver) and mapped the proteins to the genes in the microarray gene expression dataset to construct the gene interaction network. We integrated four of the microarray gene expression datasets, namely, GSE7390, GSE6532, GSE21653, and GSE11121, to increase the dataset size, while the fifth microarray dataset, namely, van de Vijver, was used as independent testing dataset. Six training sets were then generated from the integrated dataset for the extraction of the subnetwork based gene signatures, which is a set of genes that show stability not only on a specific dataset but also across multiple datasets that have distinct platforms. Two testing sets (the Desmedt (GSE7390) [20] and van de Vijver [11]) were also used for evaluating the algorithm's performance. The details are presented in the following subsections.

### 2.1. Breast Cancer Gene Expression Datasets

We used five publicly available breast cancer microarray gene expression datasets by considering the factors in the dataset, that is, estrogen-receptor status (ER+ and ER−), histologic grade (Grade 1, Grade 2, and Grade 3), overall survival (OS), and distant metastasis free survival (DMFS). In our study, 703 ER+ samples and 255 ER− samples were used for experimental analysis (selected on the basis of availability of above criterion), with a total of 958 samples. The detailed information regarding the size of the samples is shown in Table 1.

The microarray gene expression datasets for breast cancer were downloaded from the National Center for Biotechnology Information (NCBI) Gene Expression Omnibus (GEO) [24] on April 1, 2012; then, gene expression values of each dataset were normalised (or rescaled) using the formula:

(1) $$\begin{array}{c} {\hat{g}}_{n}^{i}=\frac{g_{n}^{i}-g^{\min(i)}}{g^{\max(i)}-g^{\min(i)}}, \end{array}$$

where *g*_*n*_^*i*^ defines the gene expression value of the *i*th feature in the sample *n*, *g* ^min(*i*)^ and *g* ^max(*i*)^ define the minimum and maximum gene expression values for the *i*th feature in a dataset, respectively. This normalization mapped the gene expression values generated from different protocols into a uniform framework, so that the impact of the different protocols on the data integration can be reduced. Compared with the original data, the normalized gene expressions did not show any significant differences among study objects. The datasets were converted from probe expression to gene expression, as described by Reyal et al. [25]. The probes that begin with “AFFX” are then deleted because there are no associated genes for these probes.

### 2.2. Transformation of PPI Datasets

Protein interactions play important roles in a number of biological processes where the physiological interactions of several proteins are indulged in the construction of biological pathways, such as signal transduction pathways or metabolic pathways. We incorporated six PPI datasets into our study, namely, Biological General Repository for Interaction Datasets (BIOGRID) [26], INTACT [27], the Molecular Interaction Database (MINT) [28], Database of Interacting Proteins (DIP) [16], the Biomolecular Interaction Network Database (BIND) [29], and Human Protein Reference Database (HPRD) [17]. The genes in the microarray dataset were then used to construct the gene interaction network from these PPI datasets using the Universal Protein Resource Database [30]. The self-interactions and the duplicate edges within the constructed gene interaction network were removed, as they did not have any significant meaning in terms of interaction with other genes. The resulting gene interaction network from the above mentioned six PPI datasets contains 13,012 unique genes with 69,914 unique interactions among them. All the protein interaction datasets were downloaded on April 19, 2013.

The PPI datasets contain a large number of protein interactions and are considered a rich information source from which biological knowledge and facts can be discovered, such as classifying ER+/ER− breast cancer subclasses or classifying patients according to their treatment outcome. However, the analyses of high-throughput protein interaction data signifies that protein interactions identified by experiments usually contain false-positive interactions (the interactions takes place in the experimental dataset but never happen in real biological processes or cells). It is believed that nearly 30–50% of interactions identified by experiments were biologically relevant, with few overlaps among protein interaction datasets from various resources [18]. As a consequence, discovered biological knowledge or inferred facts from the protein interaction database may be biased. Therefore, the identification and extraction of reliable protein interactions from the original published protein interaction datasets are considered an important yet challenging issue. With this attention, the quality of the protein interaction datasets can significantly improve and as a result strengthen the confidence of the discovered biological knowledge and facts. Since gene interaction networks are constructed from PPI networks, we combine three distinct reliability metrics to form a weighted reliability metric (*μ*) to measure the reliability of gene interactions. In this paper, the details are presented in Section 3.1.

### 2.3. Training and Testing Sets

To resolve the “curse of dimensionality issue,” we used five breast cancer gene expression datasets across four unique platforms in order to increase the sample size and also to balance the other factors, as mentioned in Table 1. The integrated dataset was constructed by merging four microarray datasets, namely, GSE7390, GSE6532, GSE21653, and GSE11121, which contained 958 samples. Six training sets were then constructed from the integrated dataset by initially dividing the integrated dataset on the basis of estrogen receptor status, that is, ER+ and ER−. Then, we divided the ER+ and ER− set on the basis of histologic grade, that is, Grade 1, Grade 2, and Grade 3, which led to three training sets for each estrogen receptor status, thereby, generating six training sets that were used for deriving the estrogen-receptor based gene signatures.

Sotiriou et al. [8] observed that breast cancer datasets based on histologic grades had distinct gene expression profiles. In our study, the generation of six training sets on the basis of estrogen receptor status and the histologic grade reduced the bias in the datasets and increased the correlation of gene expressions within them. The six training sets used in our algorithm constructed effective gene signatures for two estrogen-receptor subtypes of breast cancer, as presented in Section 3. The subnetwork based gene signatures generated from the training sets were then tested on two testing sets (the Desmedt dataset and the van de Vijver dataset). The results are presented in Section 4.

## 3. Algorithm

Our main focus was to extract the gene subnetworks that showed highly correlated gene expressions with the estrogen receptor status. For this the reliable gene expression metric was established to target real gene interactions that occur in biological processes and which are related to ER+/ER− breast cancers. By using the generated reliable gene expressions, the subnetwork based gene signatures that were extracted can classify ER+/ER− breast cancer patients. All the statistical validation was performed using *R* Statistical Toolbox [31]. Details of our algorithm are presented in the following subsections.

### 3.1. Reliability Metrics

For an interaction between any two genes, we combined three reliability measures to assess reliability in terms of three distinct factors, that is, data sources (e.g., HPRD), experimental methods (e.g., two hybrids), and level-based interaction partners (e.g., level-2 interaction partners of a gene). The corresponding reliability measures are named *R*_1_, *R*_2_, and *R*_3_ (data sources, experimental methods and interaction partners, resp.). These reliability measures are defined below.

#### 3.1.1. Data Source-Based Reliability (*R*_1_)

Our first reliability measure is concerned with data sources that contain protein-protein interactions and from which protein interactions are mapped to the interaction of genes. In our study, we considered data sources, such as those defined in Section 2.2. The basic aim of *R*_1_ is to evaluate the weight of gene interactions across data sources. For an interaction *y* between any two genes (*a*, *b*), *R*_1_ is calculated by counting the number of data sources that contain *y*; that is,

(2) $$\begin{array}{c} R_{1}^{\left(y\right)}=\sum_{n=1}^{S}D_{n}^{\left(y\right)}, \end{array}$$

where

(3) $$\begin{array}{c} {\;D}_{n}^{\left(y\right)}=\{\begin{array}{cc} 1, & \text{if}\;\text{data}\;\text{source}\;n\;\text{contains}\;\text{interaction}\;y,\\ 0, & \text{otherwise}. \end{array} \end{array}$$

Here, *S* defines the number of data sources. The rationale for this definition is the more data sources the interaction is regenerated in, the reliable it is. Therefore, the higher the *R*_1_ IS, the more reliable the gene interaction is.

#### 3.1.2. Experimental Method-Based Reliability (*R*_2_)

The second reliability measure evaluates the reliability of an interaction on the basis of the experimental methods. The basic idea is the same as *R*_1_; however, this time we consider how many experimental methods (e.g., affinity-chromatography, in vivo, in vitro) identified a particular interaction. Therefore, *R*_2_ is defined as the reliability measure which evaluates the reliability of any interaction *y* between (*a*, *b*) by counting the number of experimental methods that identified *y*; that is,

(4) $$\begin{array}{c} {\;R}_{2}^{\left(y\right)}=\sum_{n=1}^{N}E_{n}^{\left(y\right)}, \end{array}$$

where

(5) $$\begin{array}{c} {\;E}_{n}^{\left(y\right)}=\{\begin{array}{cc} 1, & \text{if}\;\text{experimental}\;\text{method}\;n\;\\ & \text{identified}\;\text{interaction}\;y,\\ 0, & \text{otherwise}. \end{array} \end{array}$$

Here, *N* defines the number of experimental methods. The higher the *R*_2_ of an interaction is, the more reliable the gene interaction is.

#### 3.1.3. Interaction Level-Based Reliability (*R*_3_)

The third reliability measure evaluates the reliability by considering the gene partners of two directly interacting genes. In a gene interaction network, if any two interacting genes have a higher number of level-2 neighbours (if any gene *a* is interacting with gene *b*, then *b* is level-1 neighbour of *a*, and the interaction partners of *b* are level-2 neighbours of *a*), they are considered more reliable among those with a lower number of level-2 neighbours [32, 33]. The principle behind this reliability measure is that the interacting gene pairs that interact with the genes, but which have no further interactions, are more likely to be an unreliable or false-positive interaction. However, if they have further interactions, they are seen as reliable interactions because the biological processes performed their functions in the group of interactions that is more complex compared to others.

Therefore, *R*_3_ is defined as the reliability measure that evaluates the reliability of any interaction *y* between (*a*, *b*) by counting the number of their level-2 neighbours; that is,

(6) $$\begin{array}{c} {\;\;\;\;R}_{3}^{\left(y\right)}=\sum_{n=1}^{M}\left[I_{n}^{a}+\;I_{n}^{b}\right], \end{array}$$

where

(7) $$\begin{array}{c} {\;I}_{n}^{a}=\{\begin{array}{cc} 1, & \text{if}\;\text{gene}\;n\;\text{is}\;\text{level-}2\;\text{neighbour}\;\text{of}\;\text{gene}\;a,\\ 0, & \text{otherwise}. \end{array}\; \end{array}$$

Here, *M* defines the total number of genes in the interaction network. For any interaction *y*, *R*_3_ is can be evaluated, where the higher the value of *R*_3_, the more reliable the gene interaction is and vice-versa.

#### 3.1.4. Weighted Reliability Measure

After evaluating the reliability measure based on data sources (*R*_1_), experimental methods (*R*_2_), and level-based interaction partners (*R*_3_), we performed two major steps. First, each of the reliability measures was normalised (by using the formula similar to (1)) across gene interactions, where the normalized reliabilities are within the range [0, 1]. The essentiality of normalisation is to propose a global scale of reliability that defines the reliability strength of each reliability measure within that scale. For simplicity, we still denote the normalised reliabilities as *R*_1_, *R*_2_, and *R*_3_, which were then used to construct multivariate linear regression model to form the weighted reliability measure (*μ*) defined as

(8) $$\begin{array}{c} {{\;\mu }^{\left(y\right)}=\beta }_{0}+\sum_{n=1}^{3}\beta_{n}{\;R}_{n}^{\left(y\right)}, \end{array}$$

where *μ* ^(*y*)^ defines the weighted reliability measure for *y*th interaction, *β*_0_ defines the constant, and *β*_*n*_ defines the regression coefficient for the *n*th reliability measure (i.e., *R*_*n*_^(*y*)^ variable). Here, *R*_*n*_^(*y*)^ is a promoting factor if *β*_*n*_ > 0, and *R*_*n*_^(*y*)^ is a supressing factor if *β*_*n*_ < 0. A complete model with a *P* value less than or equal to 0.05 was considered to be statistically significant.

### 3.2. Gene Expression Metrics

For each of the *k* training sets, the gene expression values of each gene are summarized by calculating the generalized mean of gene expressions (*G*) across the samples, which is defined as

(9) $$\begin{array}{c} G^{\left(a\right)}=\sqrt{\frac{1}{n}\sum_{i=1}^{n}{\left[g_{i}^{a}\right]}^{2}}, \end{array}$$

where *n* is the total number of samples, and *g*_*i*_^*a*^ defines the gene expression value of gene *a* in the *i*th sample. Next, each gene in our gene interaction network is assigned a summarized value from each training set using (9), thus leading to a total of *k* gene interaction networks (from *k* training sets). Finally, in each gene interaction network, each interaction *y* between (*a*, *b*) is then assigned a merged gene expression (*σ*) value from their interacting genes, which is defined as

(10) $$\begin{array}{c} \sigma^{\left(y\right)}=\frac{2*G^{\left(a\right)}*G^{\left(b\right)}}{G^{\left(a\right)}+G^{\left(b\right)}}. \end{array}$$

Since the gene interactions are not reliable and contain many false-positive interactions that do not take place in real biological processes (see Section 2.2), therefore, we need to combine the *σ* values and the reliability measure *μ* to accurately identify the reliable subnetwork based on differentially expressed genes.

### 3.3. Reliable Gene Expression Metrics

To construct the gene network that signifies the reliability of each gene interaction with their associated gene expressions, we incorporated the proposed reliability measure (*μ*) with the merged gene expression value (*σ*) of gene interactions and called it reliable gene expression (*θ*). As the *μ* measure assesses the reliability of each gene interaction on the basis of three vital criteria, the *σ* measure assesses the integrated gene expression of each gene interaction.

However, before defining the *θ* metric, we need to define the correlation between *μ* and *σ* to evaluate whether or not the *μ* and *σ* of any interaction *y* between (*a*, *b*) are positively correlated. We evaluate the correlation coefficient (*δ*) as

(11) $$\begin{array}{c} \delta^{\left(y\right)}=\frac{\left(\mu^{\left(y\right)}-\overset{-}{\mu }\right)\left(\sigma^{\left(y\right)}-\overset{-}{\sigma }\right)}{\sqrt{{\left(\mu^{\left(y\right)}-\overset{-}{\mu }\right)}^{2}{\left(\sigma^{\left(y\right)}-\overset{-}{\sigma }\right)}^{2}}}, \end{array}$$

where $\overset{-}{\mu }$ and $\overset{-}{\sigma }$ represent the *μ* and *σ* mean of all the interactions in a training set, respectively.

With this measure (11), the relationship between *μ* and *σ* can be evaluated, that is, whether it is positively correlated (*δ* ^(*y*)^ = 1), negatively correlated (*δ* ^(*y*)^ = − 1), or not correlated at all (*δ* ^(*y*)^ = 0). We are interested in extracting the positively correlated terms as they are more strongly related to patterns that can construct gene signatures that qualitatively classify the ER+/ER− subtypes of breast cancer. In other words, for a gene interaction *y*, if the relationship strength of their *μ* and *σ* shows *δ* ^(*y*)^ = 1, then a gene interaction has more chances of being biologically true and related to the phenotype.

Once the positively correlated interactions are extracted for each of the six training sets, the *θ* can then be evaluated. The *θ* of any interaction *y* can be evaluated by performing multivariate linear regression analysis of *μ* and *σ*, which is represented as

(12) $$\begin{array}{c} \theta^{\left(y\right)}=\beta_{0}+\beta_{1}\left(\mu^{\left(y\right)}\right)+\beta_{2}\left(\sigma^{\left(y\right)}\right), \end{array}$$

where, *β*_1_ and *β*_2_ represents the regression coefficients for *μ* ^(*y*)^ and *σ* ^(*y*)^, respectively. Once evaluated, the significant gene interactions (with *P* value <0.05) are extracted for each training set. These are then used to construct the gene signatures to classify the samples based on the ER+/ER− status. Details are presented in the following subsections.

### 3.4. Robust Reliability Based Hub Gene Expression Algorithm (*RRHGE*)

**Figure 1:**
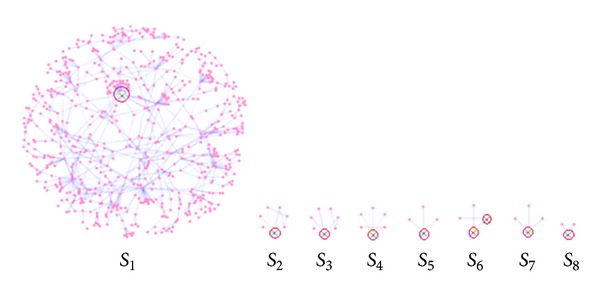
The eight subnetworks for any training set *d*. In each subnetwork, the symbol “⨂” shows the hub-gene/s, which has the highest number of interactions among other genes. In subnetwork *S*_6_, two hub-genes are identified, as they both have the maximal and equal number of interactions; that is, each gene has 2 interactions.

Significant positively correlated reliable gene interactions are used to construct the discriminative subnetworks for each training set by using the Cytoscape [34]. The *θ* values of the interactions in the discriminative subnetworks are then taken for the hub-gene evaluation, where the hub-gene is the gene in the subnetwork that contains maximal interactions amongst other genes. For each training set, it may be possible that several subnetworks exist, with each subnetwork used for the hub-gene evaluation.  Figure 1 illustrates this concept.

For each of the *k* training sets, once the subnetwork based hub-genes are identified, two major steps are performed. First, the subnetwork score (*λ*) is calculated for each subnetwork in a training set by using (13); that is,

(13) $$\begin{array}{c} {\left(\lambda \right)}_{O_{n}}=\sum_{y\in Y_{n}}\theta^{\left(y\right)}, \end{array}$$

where, *O*_*n*_  (*n* = 1,2 …, *N*) is any subnetwork, and *Y*_*n*_ is the set of all gene interactions in *O*_*n*_. A subnetwork with a maximum subnetwork score (*λ*) is chosen and retained for further analysis. A maximum *λ* based subnetwork is chosen because that subnetwork shows highly connected reliable gene interactions amongst other subnetworks for a given training set and is believed to indulge in essential real biological processes that relate to cancers. By using this step, only the subnetwork with the maximum *λ* is chosen, and other subnetworks are ignored. However, other subnetworks might contain essential genes which have the strength to be effective and stable gene signatures. Therefore, to identify those significant genes, the following operations are performed, which use hub-gene topology.

For each training set, hub-genes with their interactors for each subnetwork are identified (as shown in Figure 1). Then, the hub-gene score (*χ*) is evaluated as

(14) $$\begin{array}{c} {\left(\chi \right)}_{s_{n}}=\frac{1}{\left|H_{n}\right|}\sum_{h\in H_{n}}\theta^{\left(h\right)}, \end{array}$$

where *h* is an interaction between any gene with the hub-gene, and |*H*_*n*_| stands for the number of gene interactions that occur for the hub-gene in *O*_*n*_. In other words, for a given subnetwork, the hub-score is the average *θ* of the genes that interact with the hub-gene. In this way, the hub-score for each subnetwork in a training set can be evaluated.

The associated hub-gene score (*χ*) of the chosen subnetwork with the maximum subnetwork score (*λ*) is then used as a threshold for extracting significant gene interactions. This can be done by comparing the *χ* value of a chosen subnetwork with all of the other *χ* from other subnetworks in a given training set. If any of the other subnetworks have *χ* greater than the *χ* of the chosen subnetwork, their hub-gene with their interactors is chosen. The reason for selecting the hub-gene as the benchmark is that a hub-gene has the maximum number of interactions in a given subnetwork, with high probability these interactions will act as driver genes for cancers that indulge in several essential biological functions and processes [35]. Chang et al. [36] discovered that hub-genes are significantly related to metastasis-related genes. Also, Jonsson and Bates [37] showed that cancer-associated genes, which are translated from human proteins, show an increase in the number of interactors they interact with and also show them working as the central hubs. Therefore, hub-gene topology is used to extract the significant genes from the subnetworks (other than the chosen subnetwork) of a given training set.

Finally, for each training set, the subnetwork list, that is, a subnetwork chosen from (13) and the hub-genes with their interactors chosen from (14), is retained to extract the gene signature to classify ER+ and ER− breast cancer subtypes.  Algorithm 1 shows the pseudocode of these steps.

### 3.5. Classification Process and Performance Assessment

First, the common genes are extracted from the subnetwork lists of ER+ training sets. Operations are similar for the ER− training sets. After that, duplicates were removed between the common genes of ER+ and ER− subnetwork lists. Finally, the *RRHGE* gene signature was constructed that consists of ER+ subnetwork lists, called ER+ gene signature (for ER+ subtype) and ER− subnetwork lists, called ER− gene signature (for ER− subtype).

The *RRHGE* gene signature, which consists of ER+ and ER− gene signatures, is then used to classify ER+/ER− samples in the testing sets by transforming each sample *j* into ER+ score (*S*_+_) and ER− score (*S*_−_) using

(15) $$\begin{array}{c} S_{+}\left(j\right)=\sum_{g\in X}\frac{e\left(g,j\right)}{\left|X\right|},\\ S_{-}\left(j\right)=\sum_{k\in Y}\frac{e\left(k,j\right)}{\left|Y\right|}, \end{array}$$

where *X* = {genes of the ER+ gene signature}, *Y* = {genes of the ER− gene signature}, |•| stands for the number of set elements, and *e*(*g*, *j*) is the expression of gene *g* in sample *j*. With these two scores each sample *j* can be mapped as a point in a two-dimensional feature space ℝ^2^, then, the classification was done by three-nearest neighbour (3NN) classifier with *L*_1_ distance [38].

Since, microarray datasets usually contain unequal ratio between number of ER+ and ER− samples; therefore accuracy is not a good criteria to measure classification performance of an algorithm in the testing datasets. Rather, we used a classification performance measure, called matthews coefficient correlation (MCC) for comparison of different algorithms [39]. If TP represents the number of true positives, TN represents the number of true negatives, FP represents the number of false positives, and FN represents the number of false negatives, then the MCC can be evaluated as

(16) $$\begin{array}{c} \text{MCC}=\frac{\left(\text{TP}\times \text{TN}\right)-\left(\text{FP}\times \text{FN}\right)}{\sqrt{\left(\text{TP}+\text{FP}\right)\left(\text{TP}+\text{FN}\right)\left(\text{TN}+\text{FP}\right)\left(\text{TN}+\text{FN}\right)}}. \end{array}$$

In the above equation, if any of the four sums is 0 then the denominator is set to 1, since this results in an MCC equal to zero. In general, MCC value of 1 reflects perfect prediction, −1 reflects false prediction, and 0 reflects random prediction. MCC is a recommended measure for evaluating classification performance in comparison with other measures [38, 40].

## 4. Results

As indicated in Section 2.2, our gene interaction network contained 13,012 unique genes with 69,914 unique interactions between them, generated from six data sources. For evaluating the reliability of the gene interaction network, the weighted reliability measure (*μ*) was constructed (see (8)). The “stats” package of the *R*-project [31] has been used to evaluate the regression coefficients of *μ*; that is,

(17) $$\begin{array}{c} \mu^{\left(y\right)}=0.7138\left(R_{1}^{\left(y\right)}\right)+0.2912\left(R_{2}^{\left(y\right)}\right)+0.3072\left(R_{3}^{\left(y\right)}\right). \end{array}$$

Here, *β*_0_ is very small, that is, −4.83*E* − 15, and so we assigned *β*_0_ as zero. The complete model was significant (*P* value <0.001). With this measure (17), the higher the *μ* ^(*y*)^ value is, the higher the reliability of the interaction is.

Next, the integrated microarray dataset of 1,253 samples was constructed (see Table 1). The samples with repetitions or a missing histologic grade and estrogen receptor status based information were excluded. 958 samples remained, consisting of 703 ER+ samples and 255 ER− samples. Six training sets were then constructed from the integrated dataset, that is, three for ER+ and three for ER− (i.e., Grade 1, Grade 2, and Grade 3, resp.), which generated six gene interaction networks with their merged gene expression (*σ*) value (see Section 3.2 for details).

Further, the *μ* and *σ* were incorporated to construct reliable gene expression (*θ*) (see (12)) for each of the six training sets, by using multivariate linear regression model with their regression coefficients as shown in Table 3.

**Figure 2:**
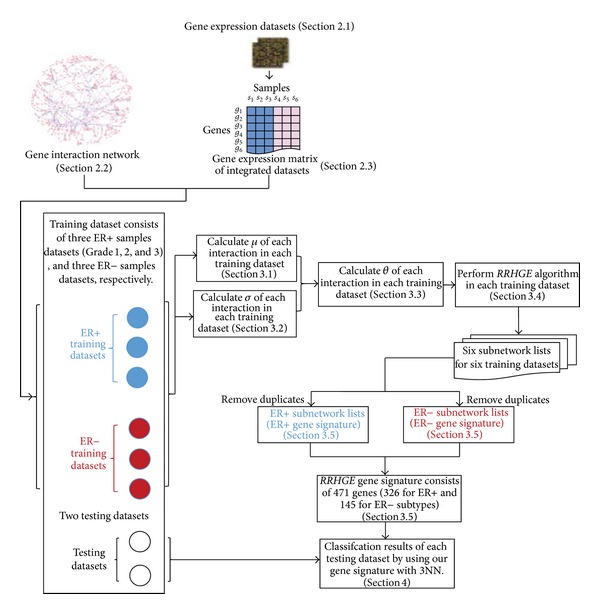
The proposed algorithm workflow. In our study, six training sets were used to generate the robust *RRHGE* gene signature, and two testing sets were used to classify the ER+/ER− breast cancer samples. The *RRHGE* gene signature set consists of 471 genes (326 for ER+ and 145 for ER− subtype).

Therefore, by applying *RRHGE* algorithm, the final gene signature set consists of 471 genes, that is, 326 distinct genes for the ER+ subtype (called ER+ gene signature) and 145 for the ER− subtype (called ER− gene signature) (see Table 2), which can classify the samples as either ER+ or ER−, as defined in Section 3.5. The complete algorithm workflow is shown in Figure 2. The Supplementary Table S1 (shown in Supplementary Material available at http://dx.doi.org/10.1155/2014/362141) lists the genes in our *RRHGE* gene signature.

The classification results on the two testing datasets, in addition to a comparison with previously established algorithms, are detailed in the following subsections.

### 4.1. Classification Performance

**Figure 3:**
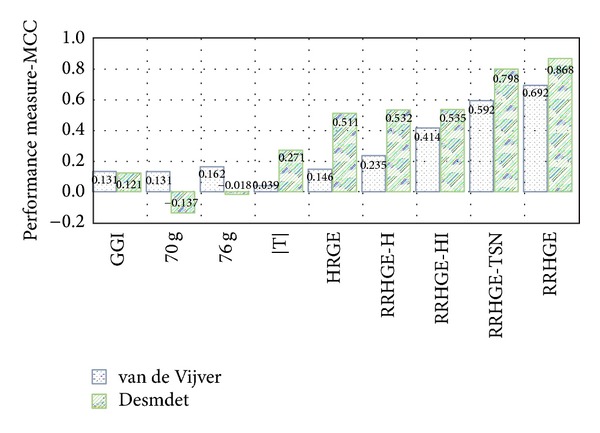
Bar charts represent the MCCs of various classification algorithms on Desmedt and van de Vijver datasets, respectively.

To test the classification performance of the gene signatures, we applied them on two testing sets, that is, Desmedt and van de Vijver datasets. We compared the *RRHGE* algorithm based on gene signature with *RRHGE-H* (i.e., gene signatures extracted from the six training sets by considering only hub-genes), *RRHGE-HI* (i.e., by considering only hub-genes with their interactions in each of the six training sets), *RRHGE-TSN* (i.e., by considering only top subnetwork in each of the six training sets), and also with five other previously existing algorithms, these are: the 70-gene signature (Mammaprint) [11], the 76-gene signature [12], the Genomic Grade Index (GGI) [8], the Interactome-Transcriptome Integration (ITI) [7], and the Hub-based Reliable Gene Expression (HRGE) [19].  Table 4 shows the detailed classification results of *RRHGE*, along with *RRHGE-H, RRHGE-HI, RRHGE-TSN*, and other existing algorithms, and Figure 3 shows the MCC comparison of algorithms in Desmedt and van de Vijver datasets, respectively. The results show that the *RRHGE* approach was able to achieve better results and was superior if considering only hub-genes in each of the six subnetworks, if considering hub-genes along with interactions in each of the six subnetworks, or if considering only top-subnetwork in each of the six subnetworks. Also, *RRHGE* is superior to other existing algorithms.

Specifically, the results show that our *RRHGE* approach achieved MCC of 0.87 and 0.70 in the testing sets of Desmedt and van de Vijver, respectively. On the Desmedt dataset, *RRHGE* gave better MCC as compared to *RRHGE-H* which gave 0.53,* RRHGE-HI* which gave 0.54, *RRHGE-TSN* which gave 0.80, HRGE which gave 0.51, ITI which gave 0.27, GGI which gave 0.12, 76 g which gave −0.02, and 70 g which gave −0.14. Amongst the existing algorithms, HRGE showed the second best MCC after *RRHGE* on Desmedt dataset. Similar results can be observed from the van de Vijver dataset, which demonstrated the *RRHGE* is still superior to other algorithms based on MCC. However, on the van de Vijver dataset, 76 g showed the second best MCC after *RRHGE*. This suggests that the classification performance of other existing gene signatures is heavily dependent on datasets and other factors, such as microarray platforms. We believe the dependency on datasets can be reduced and the classification performance can be strengthened by increasing the training compendia and incorporating multiple platforms across multiple datasets.

In the testing sets of Desmedt and van de Vijver, the HRGE, ITI, GGI, 70 g, and 76 g MCC patterns varied significantly. In other words, these algorithms were not stable enough to obtain similar classification results in distinct datasets, which indicates that these algorithms were biased towards the dataset used for classification analysis. However, the *RRHGE* subnetwork based algorithm showed stable classification performance in both testing datasets by achieving the highest MCC amongst other representative algorithms.

We also noticed that the *RRHGE-TSN* shows little lower classification performance compared with *RRHGE* (see Table 4). Therefore, if the computational costs is a concern, then *RRHGE-TSN* can serve the optimal classifier, since it requires only the top subnetwork. However, if classification performance is of the prime concern, which is the initial aim of our study, then *RRHGE* serves as the best classifier.

Since ER+ subtype generally shows higher survival rate of patients compared to ER−, so we called it a good prognosis group. However, ER− subtype shows lesser survival rate and is also more aggressive compared to ER+, so we called it a poor prognosis group [41, 42]. Now, in order to determine if the *RRHGE* gene signature is able to separate the ER+ (good prognosis) patients group and ER− (poor prognosis) patients group using the distant metastasis free survival rate (DMFS) and overall survival rate (OS) information in the microarray dataset, we performed Kaplan-Meier survival analysis.

**Figure 4:**
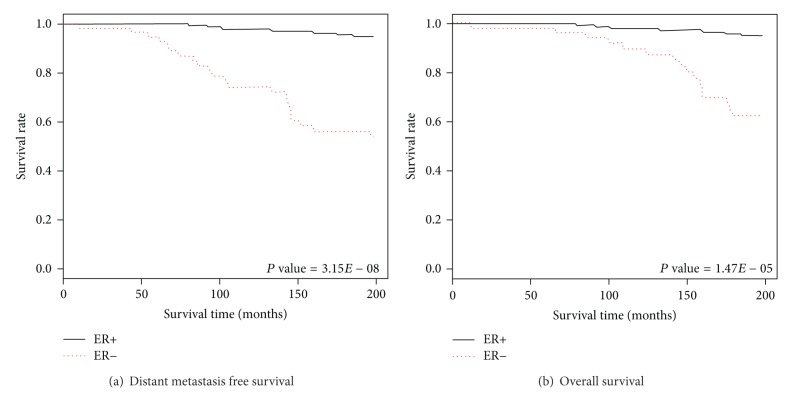
Kaplan-Meier survival graphs for ER+ and ER− patient groups in the Desmedt dataset, using the *RRHGE* gene signature (similar results achieved for van de Vijver dataset (data not shown)). A log-rank test was performed to evaluate the *P* value, which signifies that the lower the *P* value is, the better the separation between the two prognosis groups is. (a) Incorporating the DMFS rate to distinguish between ER+ or good prognosis groups (lower risk of distant metastasis) and ER− or poor prognosis groups (higher risk of distant metastasis). (b) Incorporating the OS rate that distinguishes ER+ or good prognosis groups (lower risk of death) and ER− or poor prognosis groups (higher risk of death). Both survival analysis graphs show good separation between the two prognosis groups, respectively.

The “survival” package of the *R*-project [31] has been used to perform the survival analysis between the ER+ and ER− patient groups for the Desmedt dataset, which generated the DMFS and OS survival curves of *RRHGE*, as shown in Figure 4.

We first performed the Kaplan-Meier survival analysis for distant metastasis free survival ER+ and ER− patient groups. The log-rank statistical test gave *P* value of 3.15*E* − 08, which was statistically significant (i.e., *P* < 0.001) and showed good separation between the two patient groups (Figure 4(a)). Similarly, the Kaplan-Meier survival analysis of overall survival for ER+ and ER− patient groups showed a *P* value of 1.47*E* − 05, suggesting good separation between the two patient groups (Figure 4(b)). These results validate that the *RRHGE* gene signature is effective in separating patients into two prognosis groups on the basis of the DMFS rate and the OS rate, which can determine the patient's expectancy level for the event (distant metastasis or death). Therefore, we can identify the patients group that may require more or less aggressive treatment strategy.

To illustrate the behavioural pattern of ER+ and ER− gene signatures of *RRHGE* on the Desmedt dataset, heatmaps are drawn that show the differential expressions of genes in ER+ samples and ER− samples. Although the genes in a gene signature group seem correlated with two estrogen-receptor based subtypes, no single gene shows uniformity of expressions across samples (see Supplementary Figure S1). This illustrates the significance of the gene signatures as a multigene classification method.

Using the heatmaps, distinct gene expression patterns can be visualised for the ER+ and ER− breast cancer samples. From Supplementary Figure S1, it is true to say the *RRHGE* gene signature is highly instructive in distinguishing the behavioural patterns of ER+ and ER− breast cancer subtypes.

### 4.2. Signature Stability with Existing Gene Signatures

As indicated by Garcia et al. [7], the gene signatures of van de Vijver and Wang show three mutual genes among them, which comprise less than 5% of all the genes in their signatures. We performed the gene signature stability analysis of *RRHGE* with other existing algorithms based gene signatures, as mentioned in Section 4.1. When compared with the ITI gene signature, 175 genes were found in common, corresponding to nearly 37% of genes. Compared with the 70 gene signature, only 3 genes were found in common and with the 76 gene signature, only 2 genes were found in common. In addition, the 186-gene “invasiveness” gene signature (IGS) [43] was compared with the *RRHGE* gene signature, and 10 genes were found in common. These comparison results are shown in Table 5. These small overlaps of genes signified that datasets were biased, and possibly the genes in the gene signature were not biologically relevant due to the algorithm's limitations.

We discovered that these gene signatures extracted from the subnetworks were able to achieve better results than the gene signatures extracted from the gene lists, that is, non-subnetwork based gene signatures. As ITI is a subnetwork based approach, we found that more than 35% of these genes were in common with *RRHGE*. This is significantly greater than the gene lists based gene signatures. This signifies that subnetwork based gene signatures are able to achieve higher classification performance (Table 4) and also shows higher numbers of overlapped genes amongst other gene list based gene signatures (Table 5). However, the overlap amongst subnetwork based gene signatures can be largely increased by incorporating significantly larger numbers of training sets with multiple platform types. Table 5 shows the number of genes in the *RRHGE* gene signature that overlapped with other gene signatures.

### 4.3. Biological Analysis of *RRHGE* Gene Signature

To identify the significant enriched gene ontology (GO) terms and pathways associated with the ER+ and ER− breast cancer subtypes, the enriched biological process gene ontology terms [4] and the KEGG pathways [5] were computed for each gene in our gene signature using DAVID (the Database for Annotation, Visualization, and Integrated Discovery) [44]. For each gene, the DAVID output the enriched biological process GO terms and the pathways associated with it by providing the *P* values that were computed with hyper geometric distribution. Therefore, for all genes in the gene signature, their enriched GO terms and pathways can be calculated with their *P* values in order to biologically validate the results.

First, we performed GO analysis. The Supplementary Table S2 shows the enriched GO terms for the *RRHGE* gene signature. From Table S2, it can be seen that the biological process GO terms of our subnetwork based gene signatures were correlated with the processes that were seen to be disrupted in cancers such as apoptosis, cell death, DNA damage response, insulin stimulus response, cell proliferation, nuclear mRNA splicing via spliceosome, cell cycle regulation, and many others. This demonstrates that the biological meaning of the gene signature is significant and highly associated with the cancers. The genes associated with these significant enriched GO terms are also shown in Supplementary Table S2.

Next, we performed pathway analysis. The Supplementary Table S3 shows the enriched pathways for the *RRHGE* gene signature. From Table S3, it can be seen that the pathways associated with the genes in the *RRHGE* gene signature were correlated with cancers, such as ATM signaling pathway, p53 signaling, focal adhesion class pathway, cellular aging and immortality, and many others. The genes associated with these significant enriched pathways are also shown in Supplementary Table S3.

Many of the genes in the *RRHGE* gene signature are already defined as well-known oncogenes, such as *AR, BRCA1, CDK2*, and *CCND1*, besides others, whose differential expression has been associated with the subtypes of breast cancer. For other genes not previously reported as oncogenes, these could be newly discovered genes that may act as breast cancer driver genes and may promote cancer aggressiveness by distant metastasis.

## 5. Discussions and Conclusions 

In this study, we proposed a reliable gene expression metrics (*θ*) to measure and extract the reliable gene interactions from gene interaction networks in terms of real biological processes and incorporated the extracted reliable gene interactions into our proposed algorithm: robust reliability based hub gene expression algorithm (*RRHGE*). The *RRHGE* algorithm uses hub-gene topology to identify significant genes for classifying ER+ and ER− breast cancer samples effectively. The resultant *RRHGE* gene signature consists of 471 genes, that is, 326 genes for ER+ and 145 genes for ER− subtype. From this study, we observed that the subnetwork based gene signatures are more reproducible across the datasets and are able to provide higher classification performance amongst the non-subnetwork based gene signatures.

The subnetwork based gene signature of *RRHGE* was statistically validated on the basis of MCC performance measure by comparing *RRHGE-H*,* RRHGE-HI, RRHGE-TSN*, and four other existing algorithms. The classification results (Table 4) demonstrated that our *RRHGE* based gene signature was able to accurately characterize a high number of ER+ and ER− samples in the testing sets. In other words, our gene signature was highly effective in characterizing the ER+/ER− breast cancer subtypes without depending on any specific dataset or on any other factors. The gene signature of our *RRHGE* algorithm was also validated biologically using GO and pathway analysis, and the results demonstrated that the significant enriched GO terms and pathways of the genes in our gene signature were associated with the processes shown to be disrupted in cancers.

We observed that if gene interactions in the network are reliable, then classification performance will significantly increase, compared with cases where the reliability criterion is not considered. As a matter of fact, this provides us with a possible research direction to improve the reliability metrics by incorporating highly biologically related information. In addition, it is worthwhile investigating how the classification performance behaves if we integrate other data types, such as the DNA copy number variation.

## Supplementary Material

The Supplementary Material consists of four files, including Tables S1, S2, and S3 and Figure S1. Table S1 shows the list of the genes in the gene signature constructed by the *RRHGE* algorithm. Tables S2 and S3 show the enriched gene ontology (GO) terms and the enriched pathways for the genes in the *RRHGE* gene signature, respectively, and show that the biological meaning of the *RRHGE* gene signature is significantly associated with cancers. At last, Figure S1 shows the heatmap of the *RRHGE* gene signature, which clearly shows the distinct gene expression patterns for the *RRHGE* gene signature in the ER+ and ER− breast cancer samples. These supplementary files further supports that the *RRHGE* gene signature has its advantage in classifying ER+ and ER- breast cancer patient samples effectively.

## Figures and Tables

**Algorithm 1:**
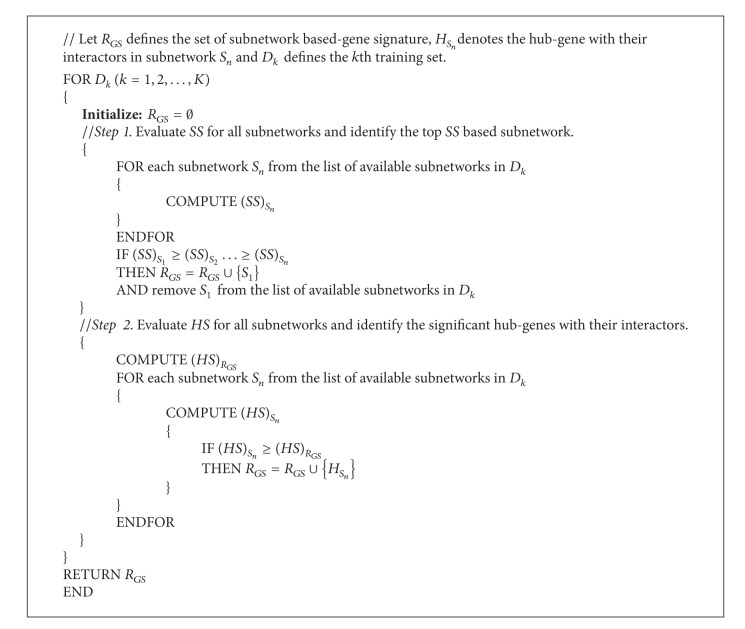
Pseudocode for the *RRHGE* algorithm.

**Table 1:** Microarray datasets used in this study.

	*Desmedt et al. [20] (GSE7390)**	van de Vijver et al. [11]**	*Loi et al. [21] (GSE6532)	*Sabatier et al. [22] (GSE21653)	*Schmidt et al. [23] (GSE11121)
Platform	HG-U133A	Agilent human genome	HG-U133A, HG-U133B	HG-U133Plus2.0	HG-U133A
Samples	198	295	327	255	200
ER					
ER+ (no. of samples)	134	226	263	150	156
ER− (no. of samples)	64	69	45	102	44
Tumour grade					
Grade 1 (no. of samples)	30	—	52	44	29
Grade 2 (no. of samples)	83	—	158	88	136
Grade 3 (no. of samples)	83	—	57	116	35
Metastasis Free Survival					
Yes (no. of samples)	62	101	70	81	46
No (no. of samples)	136	194	224	160	154
Age (in Years)					
≤40 (no. of samples)	42	—	19	49	—
41–70 (no. of samples)	156	—	241	171	—
>70 (no. of samples)	0	—	55	34	—
Average (in Years)	46	—	59	54	—

Total Samples	1253 ( 929 (ER+) and 324 (ER**−**))				

Total samples selected in our Study (on the basis of histologic grade and receptor status)	958 (703 (ER+) and 255 (ER−))				

Patients with missing histologic grade and estrogen receptor status based information are excluded from the training sets. *The datasets used in our training sets; **The testing sets.

**Table 2:** *RRHGE* gene signature size.

Training set	ER+	ER−
	Subnetwork list (no. of subnetworks)	Subnetwork list (no. of subnetworks)
Grade 1	45	31
Grade 2	35	37
Grade 3	39	34
Final gene signature set	**326**	**145**

Our gene signature set consists of 471 genes that compose 326 genes for the ER+ subtype and 145 genes for the ER− subtype.

**Table 3:** Regression coefficients of *µ* (*β*_1_) and *σ* (*β*_2_) in each of the six training sets, respectively.

Training set	*β*_0_*	*β*_1_	*β*_2_	*P* value
ER+ (Grade 1)	−8.06*E* − 16	0.4601	0.7066	<0.001
ER+ (Grade 2)	3.64*E* − 15	0.4878	0.6846	<0.001
ER+ (Grade 3)	9.81*E* − 16	0.4650	0.7094	<0.001
ER− (Grade 1)	1.49*E* − 15	0.4273	0.7274	<0.001
ER− (Grade 2)	−2.68*E* − 15	0.4484	0.7199	<0.001
ER− (Grade 3)	1.20*E* − 15	0.4673	0.7078	<0.001

*Here, *β*_0_ in each training set represents very small value and so assigned *β*_0_ as zero.

**Table 4:** Classification results of the *RRHGE* gene signature and other existing gene signatures on two testing sets, for example, (A) the Desmedt dataset and (B) the van de Vijver dataset.

	Algorithm	*N*	TP	FN	TN	FP	SN	SP	ACC	MCC
(A) Desmedt	GGI	190	84	45	29	32	0.651	0.475	0.595	0.121
	70 g	190	53	76	27	34	0.411	0.443	0.421	−0.137
	76 g	190	78	51	23	38	0.605	0.377	0.532	−0.018
	ITI	190	95	34	33	28	0.736	0.541	0.674	0.271
	HRGE	190	115	14	36	25	0.891	0.590	0.795	0.511
	RRHGE-H	190	103	26	46	15	0.798	0.754	0.784	0.532
	RRHGE-HI	190	100	29	48	13	0.775	0.787	0.779	0.535
	RRHGE-TSN	190	119	10	54	7	0.922	0.885	0.911	0.798
	RRHGE	**190**	**123**	**6**	**56**	**5**	**0.953**	**0.918**	**0.942**	**0.868**

(B) van de Vijver	GGI	150	77	37	17	19	0.675	0.472	0.627	0.131
	70 g	150	71	43	19	17	0.623	0.528	0.600	0.131
	76 g	150	72	42	20	16	0.632	0.556	0.613	0.162
	ITI	150	59	55	19	17	0.518	0.528	0.520	0.039
	HRGE	150	70	44	20	16	0.614	0.556	0.600	0.146
	RRHGE-H	146	92	22	14	18	0.807	0.438	0.726	0.235
	RRHGE-HI	150	94	20	22	14	0.825	0.611	0.773	0.414
	RRHGE-TSN	150	101	13	26	10	0.886	0.722	0.847	0.592
	RRHGE	**150**	**105**	**9**	**28**	**8**	**0.921**	**0.778**	**0.887**	**0.692**

Here, *N* defines the total number of samples, TP defines true positive (ER+ samples predicted as ER+), TN defines true negative (ER− samples predicted as ER−), FP defines false positive (ER− samples predicted as ER+), FN defines false negative (ER+ samples predicted as ER−), SE defines sensitivity, SP defines specificity, ACC defines accuracy, and MCC defines Matthews coefficient correlation. For simplicity, we represent the Genomic Grade Index as GGI, 70 gene signature as 70 g, 76 gene signature as 76 g, Interactome-Transcriptome Integration as ITI, and Hub-based Reliable Gene Expression as HRGE.. The *RRHGE* subnetwork based gene signature provides superior performance in both (A) Desmedt and (B) van de Vijver dataset.

**Table 5:** Number of overlapped genes of the *RRHGE* gene signature with ITI, 76 g, 70 g, and IGS.

	*RRHGE* overlapped genes (number (percentage))
ITI	175 (37.16%)
76 g	03 (00.64%)
70 g	05 (01.06%)
IGS	10 (02.12%)

The ITI gene signature shows the highest number of overlapping genes with the *RRHGE *gene signature, as compared to other gene signatures.
