# Supplementary material for: RRHGE: A Novel Approach to Classify the Estrogen Receptor Based Breast Cancer Subtypes
Source: ScientificWorldJournal. 2014 Jan 19;2014:362141. doi: 10.1155/2014/362141 (PMC3916021; doi:10.1155/2014/362141)

Supplementary Material

Table S1 - *RRHGE* gene signature consists of 471 genes, i.e., 326 genes for ER+ subtype and 145 genes for ER- subtype.

ER+ gene signature		
**Gene**	**ENTREZ GENE ID**	**Gene Title**
ABCB8	11194	ATP-binding cassette, sub-family B (MDR/TAP), member 8
ACIN1	22985	apoptotic chromatin condensation inducer 1
ADRB2	154	adrenergic, beta-2-, receptor, surface
AES	166	amino-terminal enhancer of split
AGR2	10551	anterior gradient homolog 2 (Xenopus laevis)
ANP32A	8125	acidic (leucine-rich) nuclear phosphoprotein 32 family, member A
AP1M2	10053	adaptor-related protein complex 1, mu 2 subunit
APEH	327	N-acylaminoacyl-peptide hydrolase
APPL1	26060	adaptor protein, phosphotyrosine interaction, PH domain and leucine zipper containing 1
APPL2	55198	adaptor protein, phosphotyrosine interaction, PH domain and leucine zipper containing 2
AR	367	androgen receptor
ARAF	369	v-raf murine sarcoma 3611 viral oncogene homolog
ARHGEF15	22899	Rho guanine nucleotide exchange factor (GEF) 15
ARL15	54622	ADP-ribosylation factor-like 15
ARL2	402	ADP-ribosylation factor-like 2
ARL2BP	23568	ADP-ribosylation factor-like 2 binding protein
ARL3	403	ADP-ribosylation factor-like 3
ARL4D	379	ADP-ribosylation factor-like 4D
ARMC7	79637	armadillo repeat containing 7
ARR3	407	arrestin 3, retinal (X-arrestin)
ASB13	79754	ankyrin repeat and SOCS box-containing 13
ATG12	9140	ATG12 autophagy related 12 homolog (S. cerevisiae)
ATG4B	23192	ATG4 autophagy related 4 homolog B (S. cerevisiae)
ATN1	1822	atrophin 1
ATP6V1G1	9550	ATPase, H+ transporting, lysosomal 13kDa, V1 subunit G1
AXIN1	8312	axin 1
BAD	572	BCL2-associated agonist of cell death
BAIAP2	10458	BAI1-associated protein 2
BAK1	578	BCL2-antagonist/killer 1
BAX	581	BCL2-associated X protein
BBC3	27113	BCL2 binding component 3
BCL2	596	B-cell CLL/lymphoma 2
BCL2L1	598	BCL2-like 1

BCL2L11	10018	BCL2-like 11 (apoptosis facilitator)
BCL2L2	599	BCL2-like 2
BECN1	8678	beclin 1, autophagy related
BIK	638	BCL2-interacting killer (apoptosis-inducing)
BIRC7	79444	baculoviral IAP repeat-containing 7
BRAP	8315	BRCA1 associated protein
BRCA1	672	breast cancer 1, early onset
BSG	682	basigin (Ok blood group)
BTRC	8945	beta-transducin repeat containing
C1orf35	79169	chromosome 1 open reading frame 35
C2orf44	80304	chromosome 2 open reading frame 44
CA12	771	carbonic anhydrase XII
CAB39	51719	calcium binding protein 39
CABIN1	23523	calcineurin binding protein 1
CALCOCO2	10241	calcium binding and coiled-coil domain 2
CASP7	840	caspase 7, apoptosis-related cysteine peptidase
CBL	867	Cas-Br-M (murine) ecotropic retroviral transforming sequence
CCDC106	29903	coiled-coil domain containing 106
CCDC33	80125	coiled-coil domain containing 33
CCDC85B	11007	coiled-coil domain containing 85B
CCND1	595	cyclin D1
CCNT1	904	cyclin T1
CCNT2	905	cyclin T2
CD81	975	CD81 molecule
CDC42	998	cell division cycle 42 (GTP binding protein, 25kDa)
CDC42EP2	10435	CDC42 effector protein (Rho GTPase binding) 2
CDK2	1017	cyclin-dependent kinase 2
CDK9	1025	cyclin-dependent kinase 9
CELSR1	9620	cadherin, EGF LAG seven-pass G-type receptor 1 (flamingo homolog, Drosophila)
CEP63	80254	centrosomal protein 63kDa
CEP76	79959	centrosomal protein 76kDa
CETN3	1070	centrin, EF-hand protein, 3
CHMP4A	29082	chromatin modifying protein 4A
CHP	11261	calcium binding protein P22
CLTC	1213	clathrin, heavy chain (Hc)
CNNM3	26505	cyclin M3
COBRA1	25920	cofactor of BRCA1
COIL	8161	coilin
COL3A1	1281	collagen, type III, alpha 1
COPS4	51138	COP9 constitutive photomorphogenic homolog subunit 4 (Arabidopsis)
COPS6	10980	COP9 constitutive photomorphogenic homolog subunit 6 (Arabidopsis)
CPSF6	11052	cleavage and polyadenylation specific factor 6, 68kDa
DAG1	1605	dystroglycan 1 (dystrophin-associated glycoprotein 1)

DAXX	1616	death-domain associated protein
DCP1A	55802	DCP1 decapping enzyme homolog A (S. cerevisiae)
DHX58	79132	DEXH (Asp-Glu-X-His) box polypeptide 58
DIABLO	56616	diablo homolog (Drosophila)
DNALI1	7802	dynein, axonemal, light intermediate chain 1
DNM1	1759	dynamin 1
DRG2	1819	developmentally regulated GTP binding protein 2
DTX2	113878	deltex homolog 2 (Drosophila)
DUSP12	11266	dual specificity phosphatase 12
E2F2	1870	E2F transcription factor 2
EDC3	80153	enhancer of mRNA decapping 3 homolog (S. cerevisiae)
EEF1A1	1915	eukaryotic translation elongation factor 1 alpha 1
EIF2B1	1967	eukaryotic translation initiation factor 2B, subunit 1 alpha, 26kDa
EIF4EBP3	8637	Eukaryotic Translation Initiation Factor 4E Binding Protein 3
ERBB3	2065	v-erb-b2 erythroblastic leukemia viral oncogene homolog 3 (avian)
FAM134A	79137	family with sequence similarity 134, member A
FAM46C	54855	family with sequence similarity 46, member C
FAM82A2	55177	family with sequence similarity 82, member A2
FAM86C	55199	family with sequence similarity 86, member C
FAU	2197	Finkel-Biskis-Reilly murine sarcoma virus (FBR-MuSV) ubiquitously expressed
FBXO34	55030	F-box protein 34
FBXW2	26190	F-box and WD repeat domain containing 2
FKBP6	8468	FK506 binding protein 6, 36kDa
FOS	2353	FBJ murine osteosarcoma viral oncogene homolog
GABARAPL2	11345	GABA(A) receptor-associated protein-like 2
GADD45GIP 1	90480	growth arrest and DNA-damage-inducible, gamma interacting protein 1
GAPDH	2597	glyceraldehyde-3-phosphate dehydrogenase
GEMIN7	79760	gem (nuclear organelle) associated protein 7
GLRX3	10539	glutaredoxin 3
GLUL	2752	glutamate-ammonia ligase
GNB2	2783	guanine nucleotide binding protein (G protein), beta polypeptide 2
GOLSYN	55638	GOLSYN A Protein
GRK6	2870	G protein-coupled receptor kinase 6
GSK3B	2932	glycogen synthase kinase 3 beta
HBXIP	10542	hepatitis B virus x interacting protein
HDAC3	8841	histone deacetylase 3
HEXIM1	10614	hexamethylene bis-acetamide inducible 1
HIF1AN	55662	hypoxia inducible factor 1, alpha subunit inhibitor
HIP1	3092	huntingtin interacting protein 1
HMOX2	3163	heme oxygenase (decycling) 2
HNRNPM	4670	heterogeneous nuclear ribonucleoprotein M
HSD17B14	51171	hydroxysteroid (17-beta) dehydrogenase 14
HSP90AB1	3326	heat shock protein 90kDa alpha (cytosolic), class B member 1

HSPB1	3315	heat shock 27kDa protein 1
HSPBP1	23640	HSPA (heat shock 70kDa) binding protein, cytoplasmic cochaperone 1
HTRA2	27429	HtrA serine peptidase 2
HUWE1	10075	HECT, UBA and WWE domain containing 1
IGF1R	3480	insulin-like growth factor 1 receptor
IGF2R	3482	insulin-like growth factor 2 receptor
IK	3550	IK cytokine, down-regulator of HLA II
IKBKAP	8518	inhibitor of kappa light polypeptide gene enhancer in B-cells, kinase complex-associated protein
IKBKE	9641	inhibitor of kappa light polypeptide gene enhancer in B-cells, kinase epsilon
INO80B	83444	INO80 complex subunit B
INVS	27130	inversin
IQGAP1	8826	IQ motif containing GTPase activating protein 1
IRS1	3667	insulin receptor substrate 1
ISYNA1	51477	inositol-3-phosphate synthase 1
ITGB3BP	23421	integrin beta 3 binding protein (beta3-endonexin)
KIAA0174	9798	KIAA0174
KIAA1279	26128	KIAA1279
KIDINS220	57498	kinase D-interacting substrate, 220kDa
KIF15	56992	kinesin family member 15
KIF22	3835	kinesin family member 22
KLC1	3831	kinesin light chain 1
KLC2	64837	kinesin light chain 2
KLHL12	59349	kelch-like 12 (Drosophila)
KLHL2	11275	kelch-like 2, Mayven (Drosophila)
LAMC1	3915	laminin, gamma 1 (formerly LAMB2)
LIG4	3981	ligase IV, DNA, ATP-dependent
LIMK1	3984	LIM domain kinase 1
LRPAP1	4043	low density lipoprotein receptor-related protein associated protein 1
LRPPRC	10128	leucine-rich PPR-motif containing
Magmas	51025	mitochondria associated protein involved in granulocyte macrophage colony stimulating factor signal transduction
MAP2K2	5605	mitogen-activated protein kinase kinase 2
MAP3K1	4214	mitogen-activated protein kinase kinase kinase 1
MAPK8	5599	mitogen-activated protein kinase 8
MAPKSP1	8649	MAPK scaffold protein 1
MAPT	4137	microtubule-associated protein tau
MBIP	51562	MAP3K12 binding inhibitory protein 1
MCL1	4170	myeloid cell leukemia sequence 1 (BCL2-related)
MDM2	4193	Mdm2 p53 binding protein homolog (mouse)
MDM4	4194	Mdm4 p53 binding protein homolog (mouse)
MEA1	4201	male-enhanced antigen 1
MED22	6837	mediator complex subunit 22
MED27	9442	mediator complex subunit 27

MED28	80306	mediator complex subunit 28
MED31	51003	mediator complex subunit 31
MOAP1	64112	modulator of apoptosis 1
MOBKL3	25843	MOB1, Mps One Binder kinase activator-like 3 (yeast)
MORF4L1	10933	mortality factor 4 like 1
MRE11A	4361	MRE11 meiotic recombination 11 homolog A (S. cerevisiae)
MRFAP1L1	114932	Morf4 family associated protein 1-like 1
MRPL44	65080	mitochondrial ribosomal protein L44
MSN	4478	moesin
MUC1	4582	mucin 1, cell surface associated
MYCBP	26292	c-myc binding protein
MYL6	4637	myosin, light chain 6, alkali, smooth muscle and non-muscle
NBN	4683	nibrin
NCOA1	8648	nuclear receptor coactivator 1
NCOA2	10499	nuclear receptor coactivator 2
NCOA4	8031	nuclear receptor coactivator 4
NCOR1	9611	nuclear receptor corepressor 1
NDC80	10403	NDC80 homolog, kinetochore complex component (S. cerevisiae)
NDEL1	81565	nudE nuclear distribution gene E homolog (A. nidulans)-like 1
NDUFB8	4714	NADH dehydrogenase (ubiquinone) 1 beta subcomplex, 8, 19kDa
NDUFV2	4729	NADH dehydrogenase (ubiquinone) flavoprotein 2, 24kDa
NEDD4	4734	neural precursor cell expressed, developmentally down-regulated 4
NFKBIA	4792	nuclear factor of kappa light polypeptide gene enhancer in B-cells inhibitor, alpha
NHEJ1	79840	nonhomologous end-joining factor 1
NME3	4832	non-metastatic cells 3, protein expressed in
NOC4L	79050	nucleolar complex associated 4 homolog (S. cerevisiae)
NPDC1	56654	neural proliferation, differentiation and control, 1
NUDT18	79873	nudix (nucleoside diphosphate linked moiety X)-type motif 18
NUDT21	11051	nudix (nucleoside diphosphate linked moiety X)-type motif 21
NUP54	53371	nucleoporin 54kDa
OGFOD2	79676	2-oxoglutarate and iron-dependent oxygenase domain containing 2
ORC2L	4999	origin recognition complex, subunit 2
ORC4L	5000	origin recognition complex, subunit 4
ORC5L	5001	origin recognition complex, subunit 5
P2RY1	5028	purinergic receptor P2Y, G-protein coupled, 1
PAFAH1B2	5049	platelet-activating factor acetylhydrolase 1b, catalytic subunit 2 (30kDa)
PAK2	5062	p21 protein (Cdc42/Rac)-activated kinase 2
PARP1	142	poly (ADP-ribose) polymerase 1
PCBD1	5092	pterin-4 alpha-carbinolamine dehydratase/dimerization cofactor of hepatocyte nuclear factor 1 alpha
PDE6D	5147	phosphodiesterase 6D, cGMP-specific, rod, delta
PDLIM7	9260	PDZ and LIM domain 7 (enigma)
PEX11B	8799	peroxisomal biogenesis factor 11 beta
PEX12	5193	peroxisomal biogenesis factor 12

PEX19	5824	peroxisomal biogenesis factor 19
PEX5	5830	peroxisomal biogenesis factor 5
PHF10	55274	PHD finger protein 10
PIH1D1	55011	PIH1 domain containing 1
PIK3R1	5295	phosphoinositide-3-kinase, regulatory subunit 1 (alpha)
PIK3R4	30849	phosphoinositide-3-kinase, regulatory subunit 4
PJA2	9867	praja ring finger 2
PLA2G15	23659	phospholipase A2, group XV
PLEKHF2	79666	pleckstrin homology domain containing, family F (with FYVE domain) member 2
PLXNB1	5364	plexin B1
POLI	11201	polymerase (DNA directed) iota
POU2F1	5451	POU class 2 homeobox 1
PPP2CA	5515	protein phosphatase 2, catalytic subunit, alpha isozyme
PPP2R1A	5518	protein phosphatase 2, regulatory subunit A, alpha
PPP2R1B	5519	protein phosphatase 2, regulatory subunit A, beta
PQBP1	10084	polyglutamine binding protein 1
PRKAR1A	5573	protein kinase, cAMP-dependent, regulatory, type I, alpha (tissue specific extinguisher 1)
PRKRA	8575	protein kinase, interferon-inducible double stranded RNA dependent activator
PRPF4	9128	PRP4 pre-mRNA processing factor 4 homolog (yeast)
PRR13	54458	proline rich 13
PSAP	5660	prosaposin
PTBP2	58155	polypyrimidine tract binding protein 2
PTPRK	5796	protein tyrosine phosphatase, receptor type, K
PXMP3	5828	Peroxisomal Membrane Protein 3 (35kD, Zellweger Syndrome)
PXN	5829	paxillin
PYCARD	29108	PYD and CARD domain containing
QARS	5859	glutaminyl-tRNA synthetase
RAB11A	8766	RAB11A, member RAS oncogene family
RAB11FIP2	22841	RAB11 family interacting protein 2 (class I)
RAB17	64284	RAB17, member RAS oncogene family
RAB2A	5862	RAB2A, member RAS oncogene family
RAB3IL1	5866	RAB3A interacting protein (rabin3)-like 1
RABGGTB	5876	Rab geranylgeranyltransferase, beta subunit
RAD50	10111	RAD50 homolog (S. cerevisiae)
RAD51	5888	RAD51 homolog (RecA homolog, E. coli) (S. cerevisiae)
RALGPS2	55103	Ral GEF with PH domain and SH3 binding motif 2
RANBP2	5903	RAN binding protein 2
RANGAP1	5905	Ran GTPase activating protein 1
RARA	5914	retinoic acid receptor, alpha
RASSF1	11186	Ras association (RalGDS/AF-6) domain family member 1
RBM4B	83759	RNA binding motif protein 4B
RBPMS	11030	RNA binding protein with multiple splicing

RCBTB2	1102	regulator of chromosome condensation (RCC1) and BTB (POZ) domain containing protein 2
RDBP	7936	RD RNA binding protein
RGS1	5996	regulator of G-protein signaling 1
RNASE6	6039	ribonuclease, RNase A family, k6
RND1	27289	Rho family GTPase 1
RNF14	9604	ring finger protein 14
ROBLD3	28956	roadblock domain containing 3
ROGDI	79641	rogdi homolog (Drosophila)
RPL11	6135	ribosomal protein L11
RPS6KC1	26750	ribosomal protein S6 kinase, 52kDa, polypeptide 1
RWDD2B	10069	RWD domain containing 2B
RXRA	6256	retinoid X receptor, alpha
S100A10	6281	S100 calcium binding protein A10
SAFB	6294	scaffold attachment factor B
SCAND1	51282	SCAN domain containing 1
SELENBP1	8991	selenium binding protein 1
SERPINA5	5104	serpin peptidase inhibitor, clade A (alpha-1 antiproteinase, antitrypsin), member 5
SETDB1	9869	SET domain, bifurcated 1
SFRS1	6426	serine/arginine-rich splicing factor 1
SIRT6	51548	sirtuin 6
SLC9A3R1	9368	solute carrier family 9 (sodium/hydrogen exchanger), member 3 regulator 1
SMAD5	4090	SMAD family member 5
SMARCD1	6602	SWI/SNF related, matrix associated, actin dependent regulator of chromatin, subfamily d, member 1
SMC3	9126	structural maintenance of chromosomes 3
SMN1	6606	survival of motor neuron 1, telomeric
SNRPB	6628	small nuclear ribonucleoprotein polypeptides B and B1
SOS2	6655	son of sevenless homolog 2 (Drosophila)
SP1	6667	Sp1 transcription factor
SP110	3431	SP110 nuclear body protein
SPARC	6678	secreted protein, acidic, cysteine-rich (osteonectin)
SRRM2	23524	serine/arginine repetitive matrix 2
STAG2	10735	stromal antigen 2
STK11	6794	serine/threonine kinase 11
STK19	8859	serine/threonine kinase 19
STMN4	81551	stathmin-like 4
SYT17	51760	synaptotagmin XVII
TAC3	6866	tachykinin 3
TADA3L	10474	transcriptional adaptor 3
TAF1	6872	TAF1 RNA polymerase II, TATA box binding protein (TBP)-associated factor, 250kDa
TAF1A	9015	TATA box binding protein (TBP)-associated factor, RNA polymerase I, A, 48kDa
TBC1D17	79735	TBC1 domain family, member 17

TCEB1	6921	transcription elongation factor B (SIII), polypeptide 1 (15kDa, elongin C)
TCEB2	6923	transcription elongation factor B (SIII), polypeptide 2 (18kDa, elongin B)
TERF1	7013	telomeric repeat binding factor (NIMA-interacting) 1
TFG	10342	TRK-fused gene
TGFB1I1	7041	transforming growth factor beta 1 induced transcript 1
TGIF1	7050	TGFB-induced factor homeobox 1
THAP1	55145	THAP domain containing, apoptosis associated protein 1
TIMM44	10469	translocase of inner mitochondrial membrane 44 homolog (yeast)
TINF2	26277	TERF1 (TRF1)-interacting nuclear factor 2
TOE1	114034	target of EGR1, member 1 (nuclear)
TOLLIP	54472	toll interacting protein
TRIM23	373	tripartite motif-containing 23
TSC1	7248	tuberous sclerosis 1
TSC22D1	8848	TSC22 domain family, member 1
U2AF2	11338	U2 small nuclear RNA auxiliary factor 2
UBA5	79876	ubiquitin-like modifier activating enzyme 5
UBE2B	7320	ubiquitin-conjugating enzyme E2B (RAD6 homolog)
ULK2	9706	unc-51-like kinase 2 (C. elegans)
UNC119	9094	unc-119 homolog (C. elegans)
VAMP4	8674	vesicle-associated membrane protein 4
VAV3	10451	vav 3 guanine nucleotide exchange factor
VDR	7421	vitamin D (1,25- dihydroxyvitamin D3) receptor
WASL	8976	Wiskott-Aldrich syndrome-like
WDYHV1	55093	WDYHV motif containing 1
WWP1	11059	WW domain containing E3 ubiquitin protein ligase 1
XIAP	331	X-linked inhibitor of apoptosis
XPA	7507	xeroderma pigmentosum, complementation group A
XRCC4	7518	X-ray repair complementing defective repair in Chinese hamster cells 4
YY1	7528	YY1 transcription factor
ZCCHC10	54819	zinc finger, CCHC domain containing 10
ZEB2	9839	zinc finger E-box binding homeobox 2
ZFP36L1	677	zinc finger protein 36, C3H type-like 1
ZFPL1	7542	zinc finger protein-like 1
ZFYVE9	9372	zinc finger, FYVE domain containing 9
ZMYND10	51364	zinc finger, MYND-type containing 10
ZNF263	10127	zinc finger protein 263
ZNF408	79797	zinc finger protein 408
ZNF580	51157	zinc finger protein 580
ZNF764	92595	zinc finger protein 764
**ER- gene signature**		
ACTN4	81	actinin, alpha 4
ALDH2	217	aldehyde dehydrogenase 2 family (mitochondrial)

ANXA3	306	annexin A3
AOF2	23028	amine oxidase (flavin containing) domain 2
APBA1	320	amyloid beta (A4) precursor protein-binding, family A, member 1
APIP	51074	APAF1 interacting protein
APP	351	amyloid beta (A4) precursor protein
ATP5C1	509	ATP synthase, H+ transporting, mitochondrial F1 complex, gamma polypeptide 1
BCR	613	breakpoint cluster region
C10orf10	11067	chromosome 10 open reading frame 10
C10orf88	80007	chromosome 10 open reading frame 88
CBLB	868	Cas-Br-M (murine) ecotropic retroviral transforming sequence b
CBX5	23468	chromobox homolog 5
CCND3	896	cyclin D3
CCT5	22948	chaperonin containing TCP1, subunit 5 (epsilon)
CD1B	910	CD1b molecule
CDC25B	994	cell division cycle 25 homolog B (S. pombe)
CKAP4	10970	cytoskeleton-associated protein 4
COPB2	9276	coatomer protein complex, subunit beta 2 (beta prime)
CSNK2A1	1457	casein kinase 2, alpha 1 polypeptide
CSNK2A2	1459	casein kinase 2, alpha prime polypeptide
CSNK2B	1460	casein kinase 2, beta polypeptide
CTSB	1508	cathepsin B
CUTC	51076	cutC copper transporter homolog (E. coli)
CXCL13	10563	chemokine (C-X-C motif) ligand 13
DDX1	1653	DEAD (Asp-Glu-Ala-Asp) box polypeptide 1
DFFA	1676	DNA fragmentation factor, 45kDa, alpha polypeptide
DFFB	1677	DNA fragmentation factor, 40kDa, beta polypeptide (caspase-activated DNase)
DIAPH1	1729	diaphanous homolog 1 (Drosophila)
DKC1	1736	dyskeratosis congenita 1, dyskerin
DOCK2	1794	dedicator of cytokinesis 2
DVL2	1856	dishevelled, dsh homolog 2 (Drosophila)
EEF1B2	1933	eukaryotic translation elongation factor 1 beta 2
EGLN1	54583	egl nine homolog 1 (C. elegans)
EIF2AK2	5610	eukaryotic translation initiation factor 2-alpha kinase 2
EIF2S1	1965	eukaryotic translation initiation factor 2, subunit 1 alpha, 35kDa
EIF3A	8661	eukaryotic translation initiation factor 3, subunit A
EIF3B	8662	eukaryotic translation initiation factor 3, subunit B
EIF4A1	1973	eukaryotic translation initiation factor 4A1
EIF4A2	1974	eukaryotic translation initiation factor 4A2
EIF4G2	1982	eukaryotic translation initiation factor 4 gamma, 2
FAS	355	Fas (TNF receptor superfamily, member 6)
FHL2	2274	four and a half LIM domains 2
FXR1	8087	fragile X mental retardation, autosomal homolog 1
FYB	2533	FYN binding protein

GADD45A	1647	growth arrest and DNA-damage-inducible, alpha
GCC1	79571	GRIP and coiled-coil domain containing 1
GOT2	2806	glutamic-oxaloacetic transaminase 2, mitochondrial (aspartate aminotransferase 2)
GPNMB	10457	glycoprotein (transmembrane) nmb
GRAP2	9402	GRB2-related adaptor protein 2
GREM1	26585	gremlin 1
GTF2H1	2965	general transcription factor IIH, polypeptide 1, 62kDa
HLA-A	3105	major histocompatibility complex, class I, A
HMG20A	10363	high-mobility group 20A
HNRNPUL1	11100	heterogeneous nuclear ribonucleoprotein U-like 1
HPCAL1	3241	hippocalcin-like 1
KARS	3735	lysyl-tRNA synthetase
KIAA0408	9729	KIAA0408
KIAA1128	54462	coiled-coil serine-rich protein 2
KIT	3815	v-kit Hardy-Zuckerman 4 feline sarcoma viral oncogene homolog
KPNA3	3839	karyopherin alpha 3 (importin alpha 4)
LAPTM5	7805	lysosomal protein transmembrane 5
LCK	3932	lymphocyte-specific protein tyrosine kinase
LCP2	3937	lymphocyte cytosolic protein 2 (SH2 domain containing leukocyte protein of 76kDa)
LMNA	4000	lamin A/C
LMNB1	4001	lamin B1
LSM6	11157	LSM6 homolog, U6 small nuclear RNA associated (S. cerevisiae)
LSM7	51690	LSM7 homolog, U6 small nuclear RNA associated (S. cerevisiae)
LTBP3	4054	latent transforming growth factor beta binding protein 3
LUM	4060	lumican
MAD1L1	8379	MAD1 mitotic arrest deficient-like 1 (yeast)
MAGOH	4116	mago-nashi homolog, proliferation-associated (Drosophila)
MAP3K5	4217	mitogen-activated protein kinase kinase kinase 5
MARK2	2011	MAP/microtubule affinity-regulating kinase 2
MBD1	4152	methyl-CpG binding domain protein 1
MBD4	8930	methyl-CpG binding domain protein 4
MCM7	4176	minichromosome maintenance complex component 7
MCOLN1	57192	mucolipin 1
MPZL1	9019	myelin protein zero-like 1
MVD	4597	mevalonate (diphospho) decarboxylase
NAE1	8883	NEDD8 activating enzyme E1 subunit 1
NCF2	4688	neutrophil cytosolic factor 2
NCK2	8440	NCK adaptor protein 2
NFYB	4801	nuclear transcription factor Y, beta
NFYC	4802	nuclear transcription factor Y, gamma
PABPC1	26986	poly(A) binding protein, cytoplasmic 1
PAFAH1B1	5048	platelet-activating factor acetylhydrolase 1b, regulatory subunit 1 (45kDa)
PAIP1	10605	poly(A) binding protein interacting protein 1

PJA1	64219	praja ring finger 1
PLS3	5358	plastin 3
PLSCR3	57048	phospholipid scramblase 3
PRNP	5621	prion protein
PRPF40A	55660	PRP40 pre-mRNA processing factor 40 homolog A (S. cerevisiae)
PSMD11	5717	proteasome (prosome, macropain) 26S subunit, non-ATPase, 11
PTCD3	55037	Pentatricopeptide repeat domain 3
RBM7	10179	RNA binding motif protein 7
RBM8A	9939	RNA binding motif protein 8A
RCN2	5955	reticulocalbin 2, EF-hand calcium binding domain
RCOR1	23186	REST corepressor 1
RELB	5971	v-rel reticuloendotheliosis viral oncogene homolog B
RGS2	5997	regulator of G-protein signaling 2, 24kDa
RNF111	54778	ring finger protein 111
RNF13	11342	ring finger protein 13
RNF146	81847	ring finger protein 146
RPA3	6119	replication protein A3, 14kDa
RPL5	6125	ribosomal protein L5
RPS15	6209	ribosomal protein S15
RUNX3	864	runt-related transcription factor 3
S100A4	6275	S100 calcium binding protein A4
SAE1	10055	SUMO1 activating enzyme subunit 1
SERPINB9	5272	serpin peptidase inhibitor, clade B (ovalbumin), member 9
SMAD1	4086	SMAD family member 1
SMURF1	57154	SMAD specific E3 ubiquitin protein ligase 1
SMURF2	64750	SMAD specific E3 ubiquitin protein ligase 2
SNIP1	79753	Smad nuclear interacting protein 1
SNRPE	6635	small nuclear ribonucleoprotein polypeptide E
SORBS2	8470	sorbin and SH3 domain containing 2
SOS1	6654	son of sevenless homolog 1 (Drosophila)
SRGN	5552	serglycin
SSR1	6745	signal sequence receptor, alpha
SUMO2	6613	SMT3 suppressor of mif two 3 homolog 2 (S. cerevisiae)
SVIL	6840	supervillin
TBK1	29110	TANK-binding kinase 1
TNFAIP3	7128	tumor necrosis factor, alpha-induced protein 3
TNFAIP8	25816	tumor necrosis factor, alpha-induced protein 8
TNFRSF1B	7133	tumor necrosis factor receptor superfamily, member 1B
TNIP1	10318	TNFAIP3 interacting protein 1
TRAF3	7187	TNF receptor-associated factor 3
TRIM27	5987	tripartite motif-containing 27
TRIM29	23650	tripartite motif-containing 29
TSPAN6	7105	tetraspanin 6
TTC19	54902	tetratricopeptide repeat domain 19

TXN	7295	thioredoxin
TYK2	7297	tyrosine kinase 2
UBA2	10054	ubiquitin-like modifier activating enzyme 2
UBE2J1	51465	ubiquitin-conjugating enzyme E2, J1 (UBC6 homolog, yeast)
UBQLN2	29978	ubiquilin 2
UPF1	5976	UPF1 regulator of nonsense transcripts homolog (yeast)
UPF2	26019	UPF2 regulator of nonsense transcripts homolog (yeast)
UPF3B	65109	UPF3 regulator of nonsense transcripts homolog B (yeast)
WAS	7454	Wiskott-Aldrich syndrome (eczema-thrombocytopenia)
WIPF1	7456	WAS/WASL interacting protein family, member 1
XPO1	7514	exportin 1 (CRM1 homolog, yeast)
YAP1	10413	Yes-associated protein 1
YIPF3	25844	Yip1 domain family, member 3

Table S2 - Enriched GO Terms for genes in the *RRHGE* gene signature.

GO	GO Description	Genes	P-Value
GO:0010941	regulation of apoptosis	RTN4, XRCC4, XIAP, NFKBIA, MLH1, TNFSF13, BCL2L1, BAK1, IGF1R, CASP3, PPP2CA, PRKRA, DIABLO, STAMBP, PPP2R1A, VAV3, LIG4, BAD, YWHAE, BRCA1, TAX1BP1, DDIT3, CDKN1A, NME3, HDAC1, BAX, RIPK1, BIK, MAPK8	681E-11
GO:0010941	regulation of cell death	RTN4, XRCC4, XIAP, NFKBIA, MLH1, TNFSF13, BCL2L1, BAK1, IGF1R, CASP3, PPP2CA, PRKRA, DIABLO, STAMBP, PPP2R1A, VAV3, LIG4, BAD, YWHAE, BRCA1, TAX1BP1, DDIT3, CDKN1A, NME3, HDAC1, BAX, RIPK1, BIK, MAPK8	916E-11
GO:0006917	induction of apoptosis	PPP2R1A, VAV3, MLH1, TNFSF13, BAD, YWHAE, BRCA1, BAK1, CDKN1A, CASP3, NME3, PPP2CA, BAX, RIPK1, PRKRA, DIABLO, BIK, MAPK8	241E-10
GO:0042127	regulation of cell proliferation	STAMBP, XRCC4, KAT2B, STK11, IGFBP7, TNFRSF13B, NFKBIA, TNFSF13, RB1, BCL2L1, LIG4, IRS1, BRCA1, CDK2, IGF1R, CASP3, CCND1, CDKN1A, HDAC1, BAX, PRKRA, MDM4, LDOC1	130E-07
GO:0006366	transcription from RNA polymerase II promoter	CCNT2, TAF1, TAF1A, CCNT1, CDK9, TBP, GTF2B, DDIT3, MED4, TAF12, GTF2F1, TCEB2, NFE2L2, PARP1	714E-09
GO:0030521	androgen receptor signaling pathway	AR, MED4, NCOA4, RB1, RNF14, BRCA1	290E-07
GO:0043549	regulation of kinase activity	CCNT2, PPP2R1A, MAPKSP1, VAV3, CCNT1, RB1, MBIP, ROBLD3, IRS1, CDC42, CCND1, CDKN1A, CASP3, HEXIM1, PPP2CA, RIPK1, GADD45G	644E-09
GO:0032868	response to insulin stimulus	IGF1R, AR, KAT2B, FBP1, APPL1, PARP1, IRS1	528E-06
GO:0045597	positive regulation of cell differentiation	XRCC4, MAPT, FOXA1, RB1, LIG4, BAD	833E-04
GO:0042770	DNA damage response, signal transduction	CCND1, MLH1, CEP63, BRCA1	475E-04
GO:0000087	M phase of mitotic cell cycle	MAD1L1, CHFR, CDC25B	612E-03
GO:0007169	transmembrane receptor protein tyrosine kinase signaling pathway	NCK2, GRB2, CTGF, NCK1, LCP2	150E-03
GO:0007155	cell adhesion	APP, CTGF, RAC1, THBS2, APBA1	890E-03
GO:0000398	nuclear mRNA splicing, via spliceosome	FUS, HNRNPH3, UPF3B, HNRNPUL1, RSRC1, PTBP1, DDX1, SNRPD2, LSM2, HNRNPR	112E-07
GO:0070647	protein modification by small protein conjugation or removal	SUMO2, UBE2M, UBA3, NEDD8, SAE1, SMURF1, CHFR, NAE1	672E-06
GO:0015931	nucleobase, nucleoside, nucleotide and nucleic acid transport	XPO1, UPF2, UPF1, UPF3B	854E-04
GO:0009967	positive regulation of signal transduction	MEN1, REL, TBK1, RAC1	510E-03

Table S3 - Enriched pathways associated with genes in the *RRHGE* gene signature.

Pathway Class	Genes	P-Value
Cell cycle	E2F1, CDKN1A, CCND1, HDAC1, GADD45G, RB1, YWHAE, CDK2, MAD1L1, YWHAZ, YWHAH, CCND3, GADD45A, CDC25B	264E-05
Apoptosis	CASP3, XIAP, RIPK1, CASP7, BAX, NFKBIA, BCL2L1, BAD,	307E-06
Focal adhesion	COL4A2, COL4A1, CCND3, ACTN4, GRB2, FYN, RAC1, THBS2, IGF1R, CDC42, CCND1, VAV3, XIAP, MAPK8, BAD, PARVA	244E-04
Adherens junction	IGF1R, CDC42, CTNNA1, CSNK2A2, CSNK2A1, ACTN4, FYN, RAC1, CSNK2B, WAS	754E-06
p53 signaling	CDKN1A, CCND1, CASP3, BAX, GADD45G, DDB2, MDM4, CDK2, CCND3, APAF1, GADD45A	641E-07
TGF-beta signaling	SMURF2, SMAD1, SMURF1, THBS2, SMURF2, SMAD1, SMURF1, THBS2	129E-03
ErbB signaling	CDKN1A, CBL, MAPK8, BAD, NCK2, GRB2, NCK1	144E-03
Insulin signaling	CBL, FBP1, MAPK8, BAD, IRS1	147E-03
Neurotrophin signaling	IGF1R, CCND1, CASP3, BAX, MLH1, MAPK8, BAD, APPL1, YWHAZ, MAP3K5, YWHAH, GRB2, RAC1	247E-06
Wnt signaling	PPP2R1A, CCND1, BTRC, PPP2CA, MAPK8, CTNNBIP1, CSNK2A2, CSNK2A1, CCND3, RAC1, CSNK2B	647E-04
ATM Signaling	CDKN1A, NFKBIA, MAPK8, BRCA1, RAD51	332E-05
Jak-STAT signaling	CCND3, GRB2, STAM, CCND1, CBL, BCL2L1	658E-03
Chemokine signaling	GRB2, RAC1, WAS, CDC42, VAV3, NFKBIB, NFKBIA	543E-03
MAPKinase Signaling	MAP3K5, GRB2, RAC1, MAP3K3, RIPK1, NFKBIA, MAPK8	492E-03
Apoptotic Signaling in Response to DNA Damage	CASP3, CASP7, BAX, BCL2L1, BAD, PARP1	523E-06
Induction of apoptosis through DR3 and DR4/5 Death Receptors	CASP3, RIPK1, CASP7, NFKBIA	718E-04
Cytosolic DNA-sensing	RIPK1, NFKBIB, NFKBIA	202E-03
Telomeres, Telomerase, Cellular Aging, and Immortality	IGF1R, PPP2CA, RB1	115E-03
Natural killer cell mediated cytotoxicity	GRB2, FYN, RAC1, HLA-A, LCP2	123E-03
CARM1 and Regulation of the Estrogen Receptor	CCND1, GTF2F1, TBP, BRCA1	455E-04

Figure S1 - The Heatmap of the *RRHGE* gene signature by using the expression values of the genes from the Desmedt dataset, and is drawn by using R statistical package (R Development Core Team., 2008). Here, each row corresponds to the genes and each column corresponds to the samples (sorted from ER+ to ER-). The Expression levels of each gene are normalized across the samples with zero mean and standard deviation equals one, where blue represents high expression levels and yellow represents low expression levels. (A) Shows the genes highly expressed in ER+ samples i.e., by applying our ER+ gene signature (326 genes) on the Desmedt dataset visualizes two groups, one is highly expressed (ER+) and other, vice-versa. (B) Shows the genes highly expressed in ER- samples i.e., by applying our ER- gene signature (145 genes) on the Desmedt dataset visualizes two groups, one is highly expressed (ER-) and other, vice-versa.

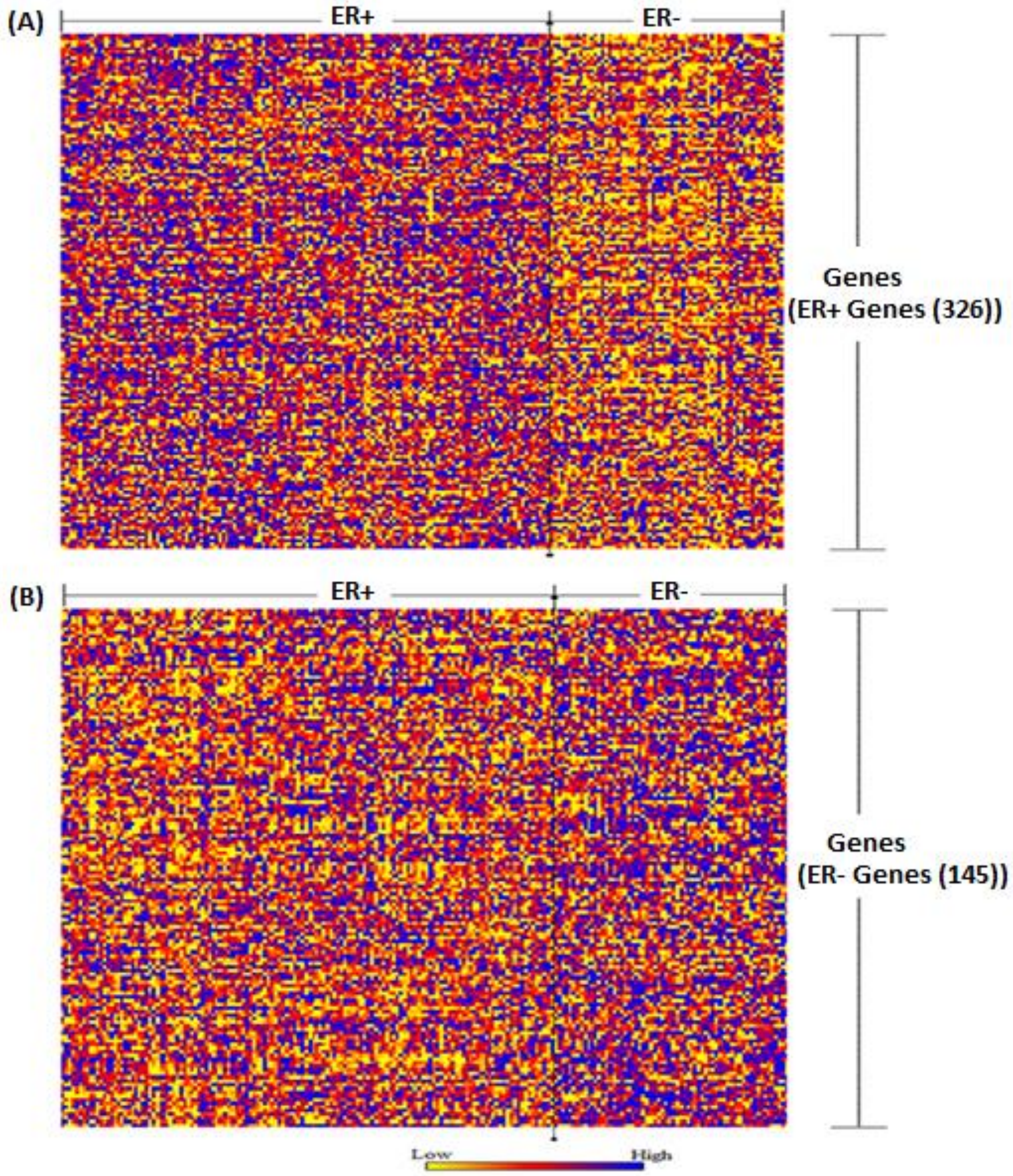

Supplement: Supplementary file 1 — The Supplementary Material consists of four files, including Tables S1, S2, and S3 and Figure S1. Table S1 shows the list of the genes in the gene signature constructed by the RRHGE algorithm. Tables S2 and S3 show the enriched gene ontology (GO) terms and the enriched pathways for the genes in the RRHGE gene signature, respectively, and show that the biological meaning of the RRHGE gene signature is significantly associated with cancers. At last, Figure S1 shows the heatmap of the RRHGE gene signature, which clearly shows the distinct gene expression patterns for the RRHGE gene signature in the ER+ and ER− breast cancer samples. These supplementary files further supports that the RRHGE gene signature has its advantage in classifying ER+ and ER- breast cancer patient samples effectively. [file 362141.f1.pdf]
